# Reproductive Pathogenic Characteristics of a Highly Virulent Porcine Reproductive and Respiratory Syndrome Virus L1J (Lineage Korean Clade C) in Gilts

**DOI:** 10.1155/tbed/1172597

**Published:** 2025-07-01

**Authors:** Jeongmin Suh, Chanhee Chae

**Affiliations:** Department of Veterinary Pathology, College of Veterinary Medicine, Seoul National University, Seoul 08826, Republic of Korea

## Abstract

Porcine reproductive and respiratory syndrome virus (PRRSV) remains a major challenge to swine health and production globally. Among PRRSV-2 lineages circulating in South Korea, the lineage 1J (L1J)—recently reclassified from lineage Korean clade C (LKC)—has emerged as an epidemiologically significant variant, accounting for approximately 15%–28.9% of cases in recent years. Despite its widespread circulation, data on the reproductive pathogenicity of L1J strains remain scarce. To address this gap, an experimental infection study was conducted to evaluate the reproductive pathogenicity of PRRSV strain SNUVR220803 in pregnant gilts. This strain, originally classified within L1J and is characterized by multiple recombination events with lineage 5 viruses-presumably the Ingelvac PRRS MLV vaccine strain, as well as a unique four-amino acid deletion in Nsp2. Eight PRRSV-naïve pregnant gilts at 86 days of gestation were randomly assigned to either the infected (*n* = 4) or control (*n* = 4) group. Inoculated gilts exhibited elevated rectal temperatures at 2 days postinoculation (dpi), followed by clinical signs including anorexia and lethargy between 7 and 10 dpi. Clinical recovery was observed by 14 dpi; however, all infected gilts subsequently experienced abortion or premature farrowing at gestational days 109–112, during which no viable piglets were recovered, except for two that died within 30 min after birth without trauma, indicating intrauterine death or severe neonatal compromise. These findings demonstrate that SNUVR220803 possesses markedly higher reproductive pathogenicity than previously reported L1J strains, such as K07-2273. Given that PRRSV reproductive virulence cannot be fully explained by ORF5-based classification alone, the heightened pathogenicity of SNUVR220803 is likely attributed to a combination of mutations in nonstructural and structural proteins. These results highlight the need for continued molecular surveillance and pathogenicity studies of emerging PRRSV strains.

## 1. Introduction

Porcine reproductive and respiratory syndrome (PRRS) is recognized as one of the leading causes of economic losses in pig production worldwide posing persistent challenges to both animal health and production efficiency. PRRS manifests as reproductive failure in pregnant sows and respiratory illness across all age groups of pigs [1]. Porcine reproductive and respiratory syndrome virus (PRRSV) is a single-stranded, positive-sense RNA virus with an envelope, classified under the family *Arteriviridae*, and the subfamily *Betaarterivirinae*. PRRSV is classified into two genetically distinct species: *Betaarterivirus suid 1* (PRRSV-1, formerly known as the European type) and *Betaarterivirus suid 2* (PRRSV-2, formerly known as the North American type) [2].

Reproductive failure in pregnant sows, including abortion, stillbirth, premature farrowing, and disrupted fetal development, is a well-recognized outcome of PRRSV infection [3]. Despite the severity of reproductive outcomes, immunological and pathological mechanisms of PRRS at the maternal-fetal interface remain poorly understood. Immunofluorescence-based investigations have recently identified the presence of PRRSV antigens and CD163-positive macrophages within the maternal and fetal areolar regions and maternal-fetal interface, indicating possible sites of viral replication and immune modulation [4]. In vitro experiments have demonstrated PRRSV-induced impairment of endometrial tight junction barrier function and reduced endometrial cell viability, implying a direct impact of the virus on uterine integrity [5].

PRRSV-2 has been particularly problematic in commercial pig farms with a prevalence rate of 57% (including 5% co-infection with PRRSV-1) of PRRSV outbreaks in South Korea [6]. To date, eleven distinct PRRSV-2 lineages have been identified globally [2], and multiple lineages are known to co-circulate within Korea. Among these, a highly virulent PRRSV strain, SNUVR220803, was recently isolated from a grower pig in Gyeongsang Province. This strain has been associated with a mortality rate exceeding 40% and marked fever, highlighting the need for further investigation into its pathogenic mechanisms. Initially classified as, linear Korean clade C (LKC) under the original PRRSV-2 Korean lineage system (clades A, B, and C) [7], the SNUVR220803 strain has since been reclassified as lineage 1J (L1J) under a refined phylogenetic framework proposed by Yim-Im et al. [2], which incorporates ORF5-based analysis and reflects genetic similarity of L1J to other lineage 1 strains.

The L1J sublineage was initially identified in South Korea in 2005 [8] and formally classified in 2018 [7]. In recent years, L1J strains have shown increased genomic complexity due to recombination with other lineages, such as lineage 11 (formerly LKA), exemplified by the emergence of recombinant strains like KNU-1901 and KNU-1902 [9]. According to a nationwide study [6], lineage 1 excluding L1J accounted for 40% of PRRSV-2 positive samples, while L1J itself comprised an additional 17% in 2022. Furthermore, government surveillance data in 2023 indicate that LKC strains represented 28.9% of PRRSV-2 cases (data not shown), highlighting the epidemiological importance of this sublineage. Despite its widespread circulation, there remains a lack of comprehensive pathogenicity data for L1J strains, particularly regarding their impact on both the respiratory and reproductive systems.

The CA-2 strain, which also belongs to the L1J lineage, has been reported to exhibit low pathogenicity in piglets [10]. In contrast, SNUVR220803 shows marked virulence in young pigs and contains several recombination regions derived from lineage 5 viruses, including the Ingelvac PRRS MLV strain. These recombination events span the 5′ untranslated region (UTR), ORF1a, and ORF1b regions-specifically, nt 39-508 (the 5′ UTR to Nsp1), nt 1254-2103 (Nsp1–Nsp2), and nt 5764-11804 (Nsp4–Nsp12)-as mapped to VR-2332 reference genome [11]. The SNUVR220803 genome also contains a unique four-amino-acid deletion within the Nsp2 region.

Although concerns about the impact of L1J strains have increased in the field, the reproductive pathogenicity of SNUVR220803 in pregnant sows has not been experimentally evaluated. Previous research has primarily focused on its respiratory and systemic effects in piglets, while clinical observations from the affected farm described increased abortion rates and a markedly reduced number of live-born pigs [11]. To date, no controlled experimental study has yet addressed the reproductive pathogenicity of highly virulent L1J PRRSV strains. Therefore, this study was conducted to evaluate the pathogenicity of the SNUVR220803 strain in pregnant gilts, with a particular focus on reproductive outcomes following experimental infection.

## 2. Material and Methods

### 2.1. Experimental Design

Eight pregnant gilts were selected and purchased from a PRRSV-free herd and confirmed negative for PRRSV by both serological testing and real-time polymerase chain reaction (qPCR). All animals were confirmed to be negative for porcine circovirus type 2 and 3 (PCV2 and PCV3) and porcine parvovirus in the serum at insemination and at arrival to exclude potential infections. Swine influenza virus, *Mycoplasma hyopneumoniae*, *Mycoplasma hyorhinis*, PCV2 and PCV3 in nasal swab were not detected by polymerase chain reaction (PCR) prior to the experiment and at arrival. Upon arrival, the animals underwent a 48-h acclimatization period before being randomly assigned to either the SNUVR220803-infected or control group (*n* = 4 per group) using a random number generator (Excel, Microsoft Corporation, Redmond, WA, USA). The number of gilts per group was limited to four to minimize environmental and temporal confounders associated with repeated animal housing. This design also ensured comparability with previous studies using a L1J strain K07-2273 [12, 13], which employed a similar number of animals. The adequacy of this group size was supported by simulation-based power analysis. The investigator responsible for data collection was blinded to the group assignments of the animals.

Gilts were housed individually in isolation facilities maintained at 25°C and allowed to farrow naturally under supervision. Gilts in the infected group received an intranasal inoculation of 6 mL of filtered cell culture supernatant containing the PRRSV strain SNUVR220803 (3^rd^ passage in MARC-145 cells, 1.2 × 10^5.0^ tissue culture infective dose 50% [TCID_50_]/mL) [14]. Control gilts were inoculated with 6 mL of sterile cell culture supernatant derived from uninfected MARC-145 cells. Inoculation was operated at 28 days before the expected farrowing date (86 days of gestation), which was an adapted experimental model from a previously described method by Terpstra et al. [15].

Colostrum samples were collected at parturition, and precolostral blood or ascitic fluid was obtained from neonates prior to suckling. Rectal temperatures of gilts were recorded daily. Live-born piglets were suckled naturally by their dams until euthanasia at 3 weeks of age. All expelled fetuses (mummified or stillborn) underwent necropsy and gross examination. Representative tissues, including thymus, lymph nodes, lungs, liver, heart, and tonsils, were collected for histopathological analysis. Birth weight and crown-rump length of neonates were measured on the day of parturition. Sows in the infected group were euthanized by electrocution and exsanguination 1 week after all fetuses or piglets were confirmed dead; sows in the control group were euthanized 3 weeks postpartum. All animal procedures were approved by the Seoul National University Institutional Animal Care and Use Committee (IACUC, SNU-230202-2-2, SNU-230830-4-2).

### 2.2. Statistical Analysis

Viral load, body weight, and crown-rump length in piglets were compared between groups using Student's *t*-test. The Mann–Whitney *U* test was applied to assess differences in nonparametric data. A *p* < 0.05 was considered statistically significant. Statistical analyses were performed using SPSS version 26.0 software (SPSS Inc., Chicago, IL, USA).

### 2.3. Pathological Analysis

Tissue samples from fetuses (lung, thymus, lymph nodes, heart, liver, and tonsils) were fixed in 10% neutral-buffered formalin for 48 h and processed for hematoxylin and eosin (H&E) staining. Lung flotation tests were conducted to differentiate stillborn from live-born piglets. Aborted fetuses were grouped into three categories based on their condition: mummified, stillborn with meconium staining, or stillborn without meconium staining.

### 2.4. Serological Analysis and PRRSV RNA Quantification

Blood samples from gilts were collected at predefined intervals: 0, 3, 7, 9, 12, 14, 18, 21, and 28 days postinoculation. Plasma samples were tested using a commercial PRRSV-specific ELISA (IDEXX, Westbrook, ME, USA), with a sample-to-positive (S/P) ratio ≥0.4 considered positive per manufacturer instructions.

RNA was extracted from plasma and tissue samples using RNAiso Plus (Takara Bio, Otsu, Japan). Quantification of PRRSV genomic RNA was performed using a real-time PCR assay (QuantStudio 1, Applied Biosystems, Foster City, CA, USA) with primers described previously [11]. A cloned target gene segment inserted into a pBHA plasmid (Bioneer, Daejeon, South Korea) was quantified spectrophotometrically to generate a standard curve. Plasmid molarity was calculated based on molecular weight and decimally diluted for standard curve construction. The assay detection limit was 153 copies/mL (Log_10_ = 2.185).

### 2.5. Plasma Cytokine ELISA

Plasma levels of IL-10, TNF-α, and IL-1β were measured using commercially available ELISA kits, following the manufacturer's protocols: Swine IL-10 ELISA Kit (KSC0101, Invitrogen, Carlsbad, CA), Porcine TNF-α ELISA Kit (ES24RB, ThermoScientific, Waltham, MA), and Porcine IL-1β ELISA Kit (ESIL1B, ThermoScientific). Plasma samples were subjected to a single freeze–thaw cycle prior to testing. Cytokine concentrations were calculated using standard curves generated by four-parameter logistic regression using MyCurveFit software (R2 v1.0.1102.823).

### 2.6. Histopathological Analysis and Immunohistochemistry

Tissue sections (3 μm) were processed for immunohistochemistry. Slides were treated with proteinase K (200× in TBST, 15 min) and 0.1% Triton X-100 for permeabilization, followed by blocking with 10% goat serum. Primary antibody against nucleocapsid (SR-30A or SDOW-17, Rural Technologies, Brookings, SD, USA) was applied, followed by incubation with alkaline phosphatase-conjugated goat anti-mouse IgG (H + L) secondary antibody (ThermoScientific) 1:1000 dilution for 2 h at 37°C. Staining was developed using the Vector Red Substrate Kit (Vector Laboratories, Mowry Ave Newark, CA).

### 2.7. Viral Genome Sequencing and Variant Analysis

Lung tissues from aborted piglets were used to isolate PRRSV by culturing in primary porcine alveolar macrophages (PAMs) free of prior PRRSV exposure. Whole-genome sequencing was then carried out using next-generation sequencing (NGS) techniques as described in earlier studies [11]. The consensus genome sequence was aligned to the reference SNUVR220803 sequence (Genbank accession no. PP074324) using MAFFT v7.310.

To assess within-host viral population diversity, high-throughput sequencing data were processed using fastp v 0.23.0 for quality control and adapter trimming. Reads with Phred scores <20 or lengths <50 bp were excluded. Filtered reads were aligned to the reference genome using BWA-MEM v0.7.18. SAM files were converted to BAM format, sorted, and indexed using Samtools v1.21. Nucleotide composition at each genomic position was analyzed using the pileup() function in the pysam v0.23.0 library. At each genomic position, the counts of A, T, C, and G nucleotides were separately tallied for reads aligned to the forward and reverse strands. Variant frequency (%) at each position was calculated as:

  $$\begin{array}{c} \text{Frequency }\left(\text{\% }\right)=\left(\frac{\text{number of reads of the specific nucleotide}}{\text{total number of reads at the position}}\right)\times \;100. \end{array}$$

## 3. Results

### 3.1. Clinical Signs of Gilts

**Figure 1:**
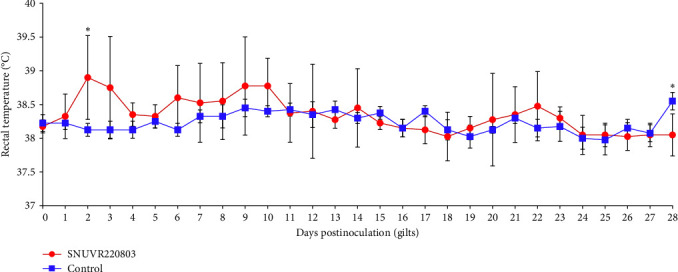
Rectal temperature of gilts. Rectal temperatures of gilts from the SNUVR220803-inoculated group and the negative control group (“SNUVR220803” and “Control,” respectively) are presented in a line graph. Group designations are indicated in the legend. Statistically significant differences (*p* < 0.05) between the two groups are denoted as asterisks.

Infected gilts exhibited inappetence and febrile responses, with a significant elevation in rectal temperature observed at 2 dpi (Figure 1). A numerical difference in rectal temperature between the SNUVR220803-inoculated gilts and the negative control group was noted at 3 dpi, after which rectal temperatures in the infected group normalized by 4 dpi.

**Figure 2:**
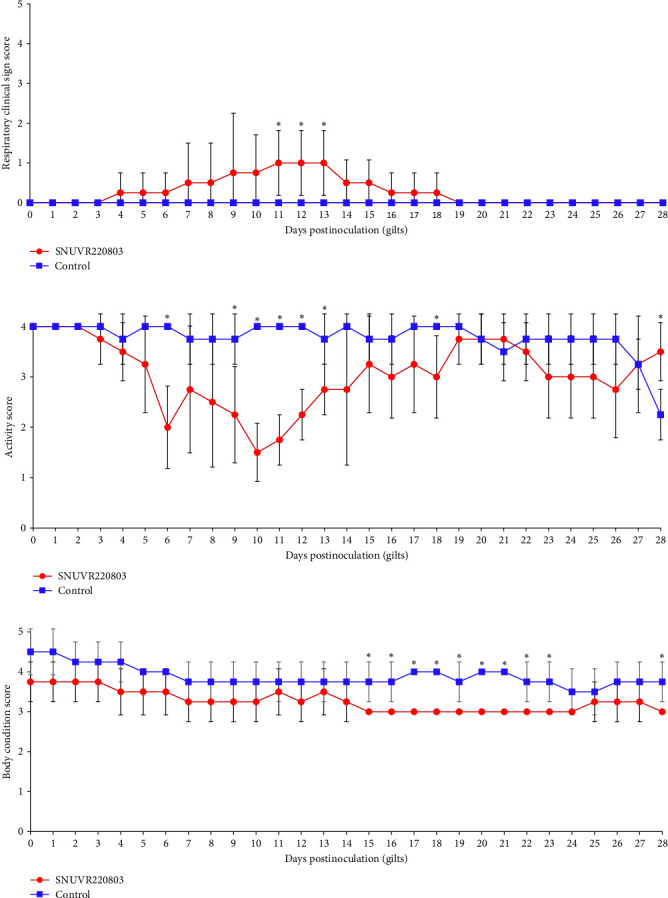
(a) Clinical sign scores, (b) activity scores, and (c) body condition scores in gilts. Respiratory clinical sign scores, activity scores, and body condition scores were evaluated in gilts from both the SNUVR220803-inoculated group and the negative control group. Each parameter is presented as a separate graph. Statistically significant differences (*p* < 0.05) between the two groups are indicated by asterisks (*⁣*^*∗*^).

Respiratory clinical scores in the infected gilts showed a noticeable increase on days 11, 12, and 13 dpi (Figure 2), following a different temporal pattern from the spike in rectal temperature. Occasional signs, such as abdominal breathing and gasping were also recorded; however, severe respiratory distress was not evident. Affected gilts often lay recumbent for extended periods and required assisted water administration, resulting in significantly reduced activity scores at 6, 9–12, 18 dpi (Figure 2 and Supporting Information: 1 Figure S1). At 28 dpi, control gilts exhibited lower activity scores, likely associated with parturition-related rest behavior.

Reduced feed intake in infected gilts was accompanied by a decline in body condition scores, which dropped by about one point between 15 and 23 dpi and remained low through 28 dpi. No additional loss in body condition was observed after 15 dpi.

### 3.2. Reproductive Outcomes

Sows in the control group farrowed normally at 114 (*n* = 3) and 115 (*n* = 1) days of gestation (Table 1). Litter sizes ranged from 13 to 15 piglets. Stillbirths per sow were 0 (*n* = 1), 1 (*n* = 2), and 3 (*n* = 1); none were PRRSV-positive by antigen testing. Eleven to twelve piglets per sow survived to weaning at 21 days of age and were subsequently euthanized for necropsy.

In contrast, all four SNUVR220803-inoculated gilts aborted between 109 and 112 days of gestation (one gilt per day) (Table 1 and Supporting Information: 2 Figure S2). Litter sizes in the infected group ranged from 14 to 16 piglets. No viable piglets were detected via lung buoyancy tests, except for two from sows that farrowed at 111 and 112 days, respectively. These two piglets exhibited weak movement at birth without vocalization but were found dead within 30 min without signs of trauma. Among the 58 fetuses from infected sows, 41.4% were stillborn without meconium staining, 31% meconium-stained, and 24.1% were mummified (Table 1).

SNUVR220803-infected sows exhibited viscous vaginal discharge for approximately 4 days, in contrast to the 1–2 days observed in the control group. Following postabortion agalactia, infected sows recovered from lethargy and inappetence within 2 days. The control sows initiated milk production and exhibited reduced activity consistent with nursing behavior until piglet weaning at 3 weeks.

### 3.3. Serological Analysis

**Figure 3:**
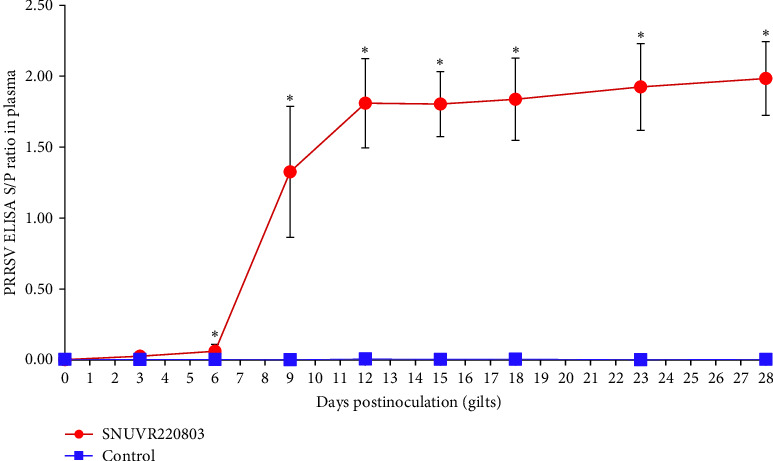
ELISA S/P ratio against PRRSV-specific antibody in gilt plasma. The sample-to-positive (S/P) ratio obtained from a PRRSV- specific was measured in plasma samples collected from gilts in the SNUVR220803-inoculated group and the negative control group. Asterisks (*⁣*^*∗*^) indicate statistically significant differences (*p* < 0.05) between the two groups.

In the SNUVR220803-infected group, ELISA S/P ratios of antibodies against PRRSV began to increase at 6 dpi and surged at 9 dpi (Figure 3). All colostrum samples from infected sows tested positive in PRRSV antibodies ELISA test (S/P ratio ≥0.4, data not shown). All piglets born to control sows were PRRSV-seronegative. Of 25 serum samples available from aborted piglets of infected gilts, 6 (24%) tested PRRSV-seropositive by PRRSV antibodies ELISA S/P ratio (≥0.4, data not shown).

### 3.4. Quantification of PRRSV RNA

**Figure 4:**
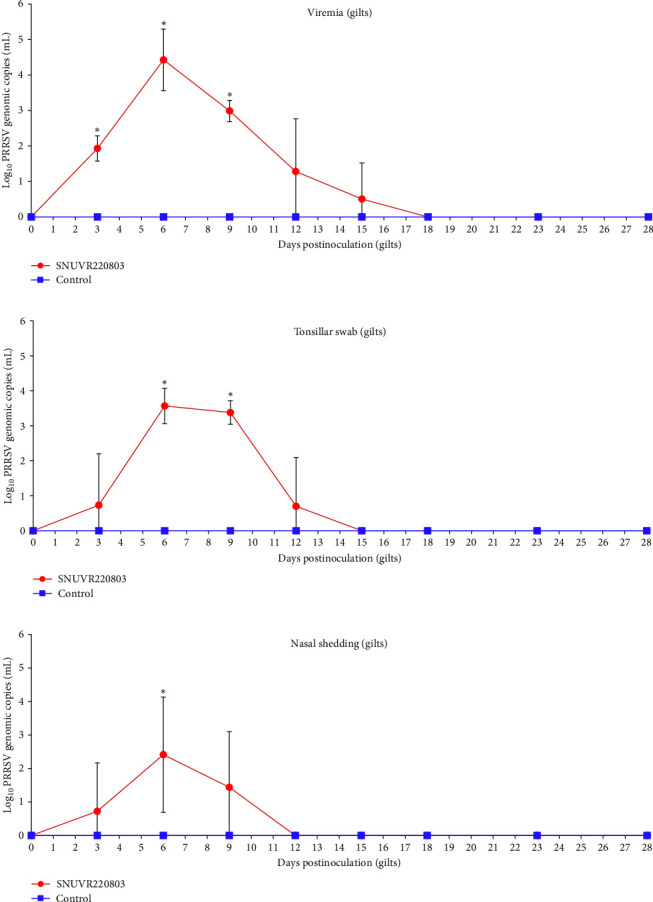
PRRSV viremia and the quantification of PRRSV in tonsillar and nasal swab of gilts. Log_10_ PRRSV genomic copies per mL were measured in (a) plasma, (b) tonsillar swab, and (c) nasal swabs from gilts in the SNUVR220803-inoculated group and the negative control group. Data are presented in graphs for each sample type. Asterisks (*⁣*^*∗*^) indicate statistically significant differences (*p* < 0.05) between the two groups.

Between 3 and 9 dpi, plasma PRRSV genomic loads were significantly higher in the infected gilts than in the controls. In addition, viral RNA levels in tonsillar and nasal swabs were also significantly elevated in the infected group at 6–9 dpi and 6 dpi, respectively (Figure 4). One infected sow (aborted at 110 days of gestation) had detectable PRRSV RNA in colostrum (10^3.32^ copies/mL); no PRRSV RNA was detected in other colostrum samples from either group. Milk secretion ceased shortly after abortion in the infected group, precluding further sampling.

**Figure 5:**
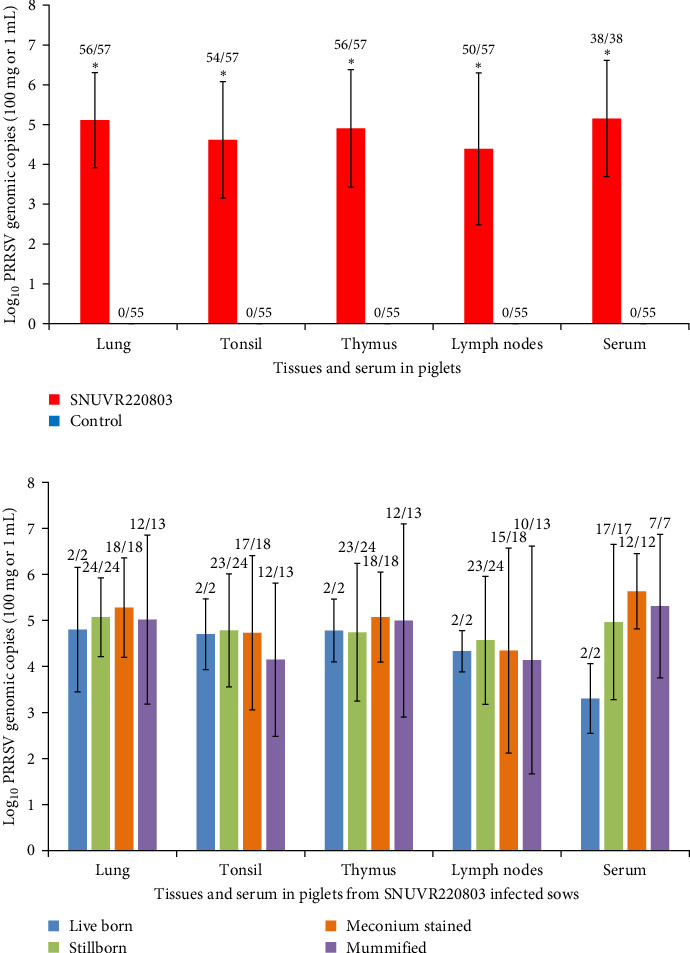
PRRSV genomic copy numbers in tissues and serum samples from newborn piglets. (a) Log_10_ PRRSV genomic copies per 100 mg of tissue (or per 1 mL of serum) from piglets born to SNUVR220803-inoculated sows and negative control sows are presented as bar graphs. Asterisks (*⁣*^*∗*^) denote statistically significant differences (*p* < 0.05) between the two groups. (b) Comparison of PRRSV genomic copy numbers among different classifications of newborns from SNUVR220803-inoculated sows: stillborn piglets without meconium staining, meconium-stained stillborn piglets, and mummified piglets. No statistically significant differences were observed among classifications (Kruskal–Wallis test). The values indicated above each bar represent the number of positive samples over the total number of samples tested (positive/total).

Viral RNA levels in lungs, tonsils, thymus, lymph nodes, and serum from newborns were significantly higher in the infected group compared to controls (Figure 5). No significant differences in viral load per tissue mass were observed across the different tissue types. Although, statistical significance was not reached among stillborn, mummified, and meconium-stained piglets within the infected group, meconium-stained individuals showed higher viral loads on average.

### 3.5. Cytokine Analysis

**Figure 6:**
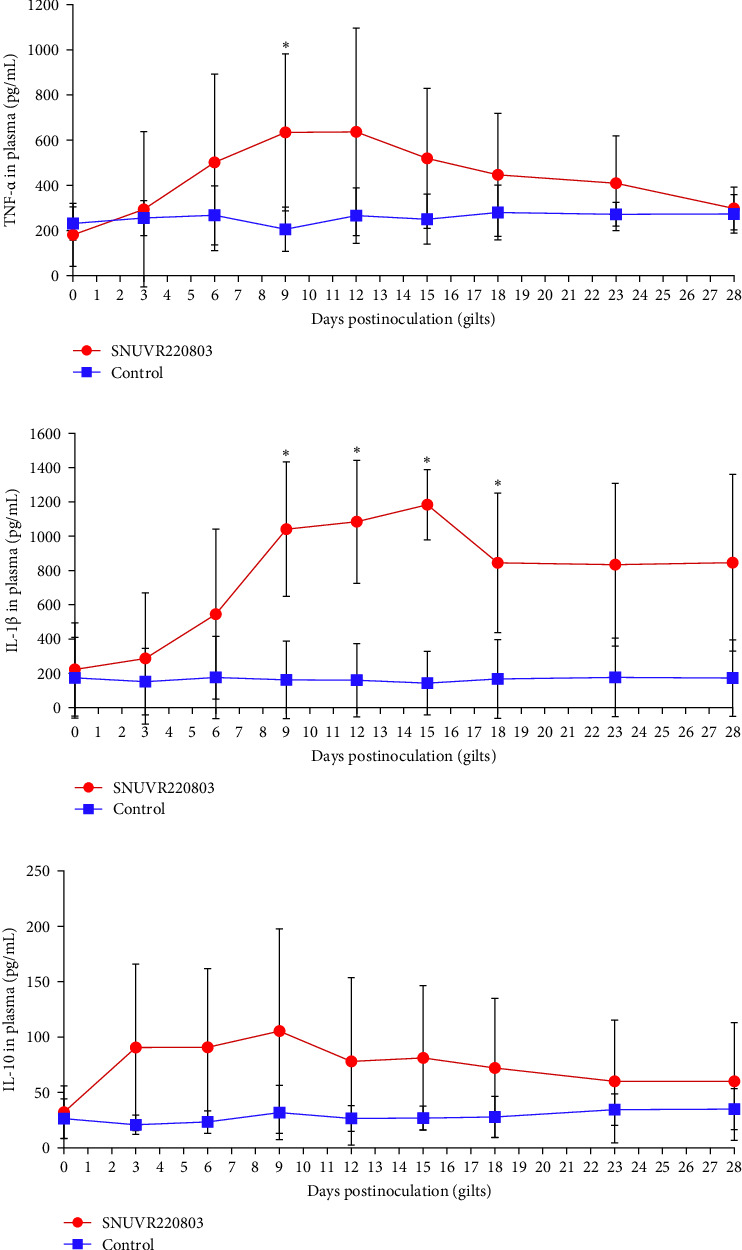
Plasma cytokine profile in gilts. Plasma cytokine profile in gilts following SNUVR220803 inoculation. Graphs show the concentrations of (a) tumor necrosis factor-α (TNF-α), (b) interleukin-1 β (IL-1β), and (c) interleukin-10 (IL-10) in the plasma samples collected at different days postinoculation from gilts in the SNUVR220803 inoculated group and the negative control group. Asterisks (*⁣*^*∗*^) indicate statistically significant differences (*p* < 0.05) between the two groups.

Plasma concentrations of TNF-α, IL-1β, and IL-10 were measured and expressed in pg/mL (Figure 6). TNF-α levels were significantly elevated in the infected group at 9 dpi and remained elevated through 12 dpi, declining by 28 dpi. IL-1β levels in infected gilts were significantly higher than controls from 9 to 18 dpi and remained numerically elevated through 28 dpi. While IL-10 concentrations did not differ significantly between groups, the infected gilts maintained consistently higher mean IL-10 levels (approximately twice those of controls) from 3 to 18 dpi.

### 3.6. Histopathological Analysis

**Figure 7:**
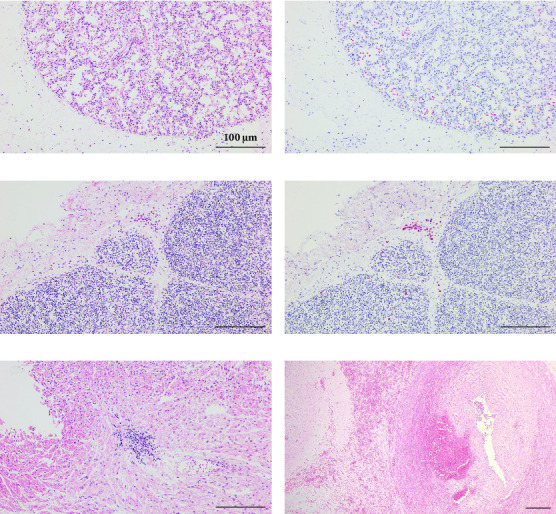
Histopathological findings in hematoxylin and eosin (H&E) and immunohistochemical staining of tissues from aborted piglets. (a, b) Serial sections of lung tissue from a fetus aborted by a sow inoculated with the SNUVR220803 strain. (a) H&E staining reveals mild thickening of alveolar septa characterized by infiltration of inflammatory cells. (b) Immunohistochemistry with SDOW-17 monoclonal antibody specific for PRRSV nucleocapsid (N) protein highlights positive signals in porcine alveolar macrophages (PAMs). (c, d) Sections of thymus. (c) H&E staining shows focal aggregation of macrophages in the connective tissue. (d) Immunohistochemistry demonstrates strong PRRSV N protein expression in macrophages within both the interstitial areas and thymic follicles. (e) H&E staining of myocardium reveals interstitial infiltration of inflammatory cells between muscle fibers. (f) H&E staining of umbilical cord shows hemorrhage both within and surrounding the vessel wall.

Histological examination of lung tissues from newborn piglets delivered by SNUVR220803-infected sows revealed mild thickening of the alveolar septa and infiltration of lymphocytes (Figure 7). In the thymus, focal aggregation of PRRSV-infected macrophages was observed within the connective tissue, accompanied by mild lymphoid depletion in thymic follicles. In addition, infiltration of inflammatory cells was noted between myocardial fibers and hemorrhagic lesions were observed in near the umbilical artery of piglets from infected sows (Figure 7). Due to extensive mummification in many aborted fetuses, the degree of thymic lymphoid depletion could not be quantified in all cases; however, depletion was qualitatively confirmed in multiple samples.

**Figure 8:**
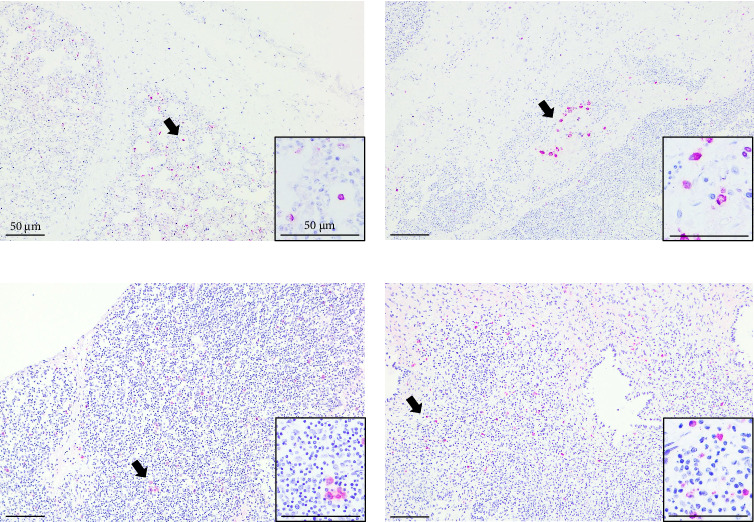
PRRSV SR-30A immunohistochemistry in the aborted piglets. Detection of PRRSV antigen in formalin-fixed, paraffin-embedded sections of (a) lung, (b) thymus, (c) lymph node, and (d) tonsil tissue using immunohistochemistry with the SR-30A monoclonal antibody, which targets the nucleocapsid protein of PRRSV. Positive signals appear as red staining, visualized using Vector Red chromogen in an alkaline phosphatase-based reaction. Black arrows indicate the PRRSV-positive cells. All images are shown at ×100 magnification; insets display representative areas at ×200 magnification.

PRRSV antigens were detected by immunohistochemistry in the lungs, thymus, lymph nodes, and tonsils of piglets from the infected group (Figure 8). Pulmonary alveolar macrophages exhibited strong PRRSV-positive signals. In the tonsils and lymph nodes, PRRSV-positive immunostaining was particularly noted in macrophages located within lymphoid follicles, interspersed among lymphocytes.

### 3.7. Sequence Analysis of Isolated PRRSV From the Lung of an Aborted Piglet

Whole-genome sequencing of PRRSV isolated from the lung of an aborted piglet (via culture in PAMs cells) was conducted and aligned to the reference SNUVR220803 genome (GenBank accession no. PP074324). Mutations were recorded as changes in consensus nucleotide sequences relative to the reference, and the variant population frequencies were summarized in Table 2.

A total of 11 nucleotide mutations were identified, of which 4 resulted in consensus changes, while the remainder introduced ambiguous positions. Among these, 8 were nonsynonymous mutations affecting viral proteins, including Nsp2 V477G and K654E, Nsp11 F186L, Nsp12 D48G, GP2 V97I, GP3 V23F, and GP4 F114L. The sequences of ORF5, ORF6, and ORF7 in the isolated strain were fully conserved relative to the reference strain.

## 4. Discussion

In contrast to a previous study involving 3-week-old piglets inoculated with the SNUVR220803 [11], infected sows exhibited a shorter viremic phase, lasting approximately 12 days, and no mortality was observed throughout the experimental period. Respiratory clinical scores in infected gilts were generally lower than those reported in weaned piglets, and symptoms subsided by 19 days postinoculation. The pathogenicity of PRRSV is known to vary depending on viral strain, host age, and immune status [16]. Age-dependent differences in PRRSV pathogenicity have been frequently reported, with sows and piglets exhibiting distinct clinical response. For example, infection with the PRRSV lineage 5 strain JA142 in sows has been associated with milder disease manifestations and a shorter duration of viremia compared to young pigs, typically lasting fewer than 14 days postinoculation [16]. These findings suggest that the milder respiratory signs observed in SNUVR220803-gilts may be attributed to the age dependency of PRRSV pathogenicity.

In the present study, PRRSV-specific antibodies were detected in the aborted fetuses, and the virus was isolated from their lungs and identified as a mutated form of SNUVR220803. No coinfection with PCV2 and PCV3, influenza A virus, porcine parvovirus, or other opportunistic pathogens was detected, supporting that abortion was likely induced solely by PRRSV SNUVR220803.

Experimental results on PRRSV-induced reproductive failure in sows vary across PRRSV-1 and PRRSV-2 strains. A previous study on PRRSV-1 demonstrated that sows infected 30 days prior to the expected farrowing date (i.e., at 84 days post artificial insemination) exhibited an abortion event in one out of eight inoculated sows at 109 days of gestation, while the remaining sows farrowed between 113 and 117 days with a high stillbirth rate [15]. Otherwise, Han et al. [14] reported that PRRSV-1 strain SNUVR100748 caused abortion in all four infected sows (100%) between 106 and 111 days of gestation, and 84.21% (32/38) of the newborns were stillborn or mummified. Similarly, PRRSV-2 strains also vary in their reproductive pathogenicity. The lineage 5 strain K08-1054 caused abortion at 112 days of gestation in one of two inoculated sows, with only 36% (9/25) of fetuses born alive [13]. In another case, the SNUVR090851 strain, whose lineage remains undefined, caused early farrowing (104–108 days) in all six inoculated sows (100%), with only 19.7% of piglets born alive [17].

Compared to these findings, the SNUVR220803 strain shows remarkably high reproductive pathogenicity, with all four inoculated sows (100%) aborting between 109 and 112 days of gestation. Among the 58 fetuses recovered from the infected group, 56 were either stillborn or mummified, indicating a fetal viability rate of only 3.4%. In contrast, the L1J (LKC) strain K07-2273, also the mother strain in the recombination event of SNUVR220803, showed milder reproductive outcomes in a previous study, with no abortion and a stillbirth rate of 66.67% (8/12) in one out of two sows [12]. In another study, one of two sows inoculated with the K07-2273 strain aborted at 107 days of gestation, and among the 19 fetuses from both sows, only 4 (21.05%) were born alive [13].

PRRSV antigens were detected in the lungs, tonsils, lymph nodes, and thymus of aborted piglets. Histopathological examination revealed alveolar wall thickening in the lungs and thymic lymphoid depletion in some cases. Notably, antigen positivity in fetal lung tissues was more prominent in pulmonary alveolar macrophages than in septal macrophages. Serum viral genomic loads in piglets born from SNUVR220803-infected sows exceeded peak maternal viremia levels. PRRSV genomes were identified in 98.2% (56/57) of piglet lungs, a markedly higher rate than the 78.95% (15/19) positivity observed in fetal lungs from K07-2273-infected sows in a previous study [13]. This suggests that SNUVR220803 has enhanced capacity for transplacental infection and viral replication in fetuses. Given the established link between high fetal viral load and pathogenicity [18], these findings may explain the severe reproductive outcomes associated with this strain.

PRRSV is known to induce the anti-inflammatory cytokine IL-10 [19], which suppresses IFN-*γ* and other proinflammatory mediators [20]. IL-10 also inhibits the expression of IL-1α, IL-6, and TNF-α, primarily through the regulation of pulmonary septal macrophages [21]. PRRSV induces IL-10 production through viral components, such as Nsp1 [22] and GP5 [23], and upregulates IL-10 in macrophages [24] and dendritic cells [25], contributing to increased monocyte susceptibility [26] and suppressing antigen-specific IFN-*γ* responses in peripheral blood mononuclear cells (PBMCs) [27]. In this study, plasma IL-10 levels increased in three gilts between 3 and 9 dpi; however, statistical significance was not reached, likely due to individual variation and the small sample size.

Despite this early anti-inflammatory response, TNF-α and IL-1β levels increased markedly from 9 dpi onward in SNUVR220803-infected gilts. TNF-α plays a key role in antiviral defense, lymphocyte recruitment, and apoptosis regulation [28], but is actively suppressed by PRRSV [22]. For example, NSP1α and NSP1β inhibit TNF-*α* transcription by modulating NF-*κ*B and Sp1 promoter activity [29], while HP-PRRSV strains have further attenuate TNF-α release via ERK pathway inhibition [30] downstream of TLR signaling [31]. Nonetheless, elevated IL-1β [32] and TNF-α [33] levels have been reported in the later stages of infection with virulent PRRSV strains, and HP-PRRSV is known to induce systemic cytokine responses, including TNF-α, IFN-α, IL-1, and IL-6, around 7 dpi [34]. Secondary peaks of TNF-α and IL-1β at 10 or 21 dpi in HP-PRRSV infection have also been observed [35]. Additionally, IL-1β expression has been shown to persist up to 28 dpi in pigs with persistent PRRSV infection compared to early virus clearers [36], and TNF-α levels have been found to be significantly higher in pigs infected with highly pathogenic strains than with low-pathogenic ones [37]. Although the precise role of IL-1β in PRRSV pathogenesis remains unclear, it may contribute to IL-6 induction [32] and neutrophil recruitment [35]. Taken together, the increased expression of IL-1β and TNF-α observed in this study may be linked to inflammatory damage at the maternal–fetal interface, contributing to reproductive pathologies, such as fetal death and abortion.

Genetically, the SNUVR220803 virus isolated from the lungs of aborted piglets possesses several mutations that may underline its phenotype adaptation. Nsp2, a component of the membrane-spanning domain essential for double-membrane vesicle formation [38], harbors V477G and K645E substitutions within its hypervariable region, which is implicated in PAM tropism, particularly in strains with deletions [39]. The Nsp11, a nidovirus-specific endoribonuclease [40], exhibits IFNβ antagonist activity independent of its endonuclease function [22] and, in this strain, carries the F141L substitution. NSP12, with a D123G mutation, is involved in subgenomic RNA synthesis [41]. Mutations in structural proteins include GP2 V97I in the ectodomain, which may influence CD163 interaction, GP3 mutations V23F (N-terminal hydrophobic region) and A93T, and the GP4 F114L, all occurring outside known antigenic epitopes [42]. Collectively, mutations in GP2, GP3, and GP4, components of the viral CD163-binding complex [43], may enhance viral entry or immune evasion [44].

SNUVR220803, initially isolated in 2022 from a grower pig in Gyeongsang province, South Korea, was linked to catastrophic reproductive failure on farms. Experimental infection in PRRSV-naïve sows confirmed complete abortion without viable offspring. Although belonging to the L1J (LKC) lineage, previously unassociated with the severe reproductive disease, SNUVR220803 demonstrated markedly enhanced virulence. These findings highlight the need for molecular and reverse genetics studies to elucidate the genomic features responsible for the elevated pathogenicity of this variant.

## 5. Conclusions

The SNUVR220803 strain caused moderate respiratory symptoms but severe reproductive failure in PRRSV-naïve sows, with a 100% abortion rate and widespread fetal infection. Although classified within the L1J (LKC) lineage, this strain demonstrated greater reproductive virulence than previously reported L1J variants. These findings highlight the possibility of pathogen variation even within established lineages and support the continued need for genomic monitoring and mechanistic research to better understand the molecular basis of PRRSV virulence.

## Figures and Tables

**Table 1:** Litter characteristics.

Group of gilts	*n* (gilts)	Gestation days	Litter characteristics			
			State at the birth	*n* (piglets)	Body weight (mean ± SD, g)	Body length (mean ± SD, cm)
SNUVR220803 Infected	4	109–112	Live born	2	1120 ± 57	29.5 ± 0.4
			Stillborn (unstained)	24	922 ± 179	27.5 ± 2.1
			Meconium stained	18	844 ± 202	26.4 ± 1.7
			Mummified	14	489 ± 85	21.9 ± 1.6
			Total	58	800 ± 247*⁣*^*∗*^	25.7 ± 2.9*⁣*^*∗*^

Control	4	114–115	Live born	50	1198 ± 186	29.6 ± 1.5
			Stillborn (unstained)	5	878 ± 135	27.3 ± 0.3
			Meconium stained	—	—	—
			Mummified	—	—	—
			Total	55	1169 ± 203*⁣*^*∗*^	29.4 ± 1.6*⁣*^*∗*^

*Note*: The body length means the crown-to-rump length of newborn piglets (0 days of age). Statistically significant differences (*p* < 0.05) between the two groups are shown as asterisks (*⁣*^*∗*^).

**Table 2:** Variation detected in the whole genome sequence of SNUVR220803 isolated from an aborted piglet and the comparison based on the original sequence of SNUVR220803 (GenBank no. PP074324).

The mutated nucleotide position	The amino acid position	Consensus nucleotides^a^		Amino acid change		Forward/reverse read numbers (percentage of reads)		The change of codon	The type of the mutation
		Original SNUVR220803	Isolated SNUVR220803	Original SNUVR220803	Mutated SNUVR220803	Original nucleotide	Mutated nucleotide		
ORF1a (1563)	NSP2 (aa 138)	C	Y	Pro	Pro	12/53 (10.02%)	136/448 (89.98%)	Synonymous	Transition

ORF1a (2579)	NSP2 (aa 477)	T	K	Val	Gly	52/51 (31.99%)	129/90 (68.01%)	Nonsynonymous	Transversion

ORF1a (3082)	NSP2 (aa 645)	A	G	Lys	Glu	8/5 (3.90%)	140/180 (96.10%)	Nonsynonymous	Transition

ORF1a (4908)	NSP3 (aa 192)	C	A	Gly	Gly	14/39 (26.63%)	72/73 (72.86%)	Synonymous	Transversion

ORF1b (3799)	NSP11 (aa 186)	T	C	Phe	Leu	−(0%)	164/119 (100%)	Nonsynonymous	Transition

ORF1b (4055)	NSP12 (aa 48)	A	R	Asp	Gly	45/50 (33.57%)	78/110 (66.43%)	Nonsynonymous	Transition

ORF2a (289–291)	GP2 (aa 97)	GTG	RTR	Val	Ile	289^th^: 39/34 (34.93%), 291^th^: 39/33 (34.12%)	289^th^: 73/63 (65.07%), 291^th^: 74/68 (65.88%)	Nonsynonymous	Transition

ORF2a (690) (ORF3 [67])	GP2 (aa 230) (GP3 [aa 23])	G	T	Val (Val)	Val (Phe)	5/13 (5.42%)	129/158 (94.58%)	Synonymous (Nonsynonymous)	Transversion

ORF3 (277)	GP3 (aa 93)	G	R	Ala	Thr	89/94 (56.13%)	75/68 (43.87%)	Nonsynonymous	Transition

ORF4 (340)	GP4 (aa 114)	T	Y	Phe	Leu	124/89 (66.98%)	56/49 (33.02%)	Nonsynonymous	Transition

^a^Ambiguous nucleotides symbols mean: R = A or G, Y = C or T, and K = G or T.

## Data Availability

Data sets used and/or analyzed during this study can be obtained from the corresponding author upon reasonable request.

## Supporting Information

Supporting Information 1 Figure S1: Clinical appearance of sows from SNUVR220803-inoculated and negative control groups. Photographs of three representative sows (two from the SNUVR220803-inoculated group and one from the negative control group) taken at various days postinoculation (dpi). (a–e) A sow that aborted at 110 days of gestation. (f–j) A sow that aborted at 111 days of gestation. (k) Nasal inoculation method in a gilt under physical restraint. (l, m) Sow from the negative control group. (a, f) at 0 dpi; (b, g) at 7 dpi; (c, h) at 14 dpi; (d, i, l) at 21 dpi; and (e, j, m) at the day of delivery (24, 25, and 28 dpi, respectively).

Supporting Information 2 Figure S2: Gross appearance of expelled or stillborn piglets from SNUVR220803-inoculated gilts. Photographs show aborted or stillborn piglets at (a) 109, (b) 110, (c) 111, and (d) 112 days of gestation.
