# Supplementary figures and images for: Reproductive Pathogenic Characteristics of a Highly Virulent Porcine Reproductive and Respiratory Syndrome Virus L1J (Lineage Korean Clade C) in Gilts
Source: Transbound Emerg Dis. 2025 Jul 1;2025:1172597. doi: 10.1155/tbed/1172597 (PMC12237560; doi:10.1155/tbed/1172597)

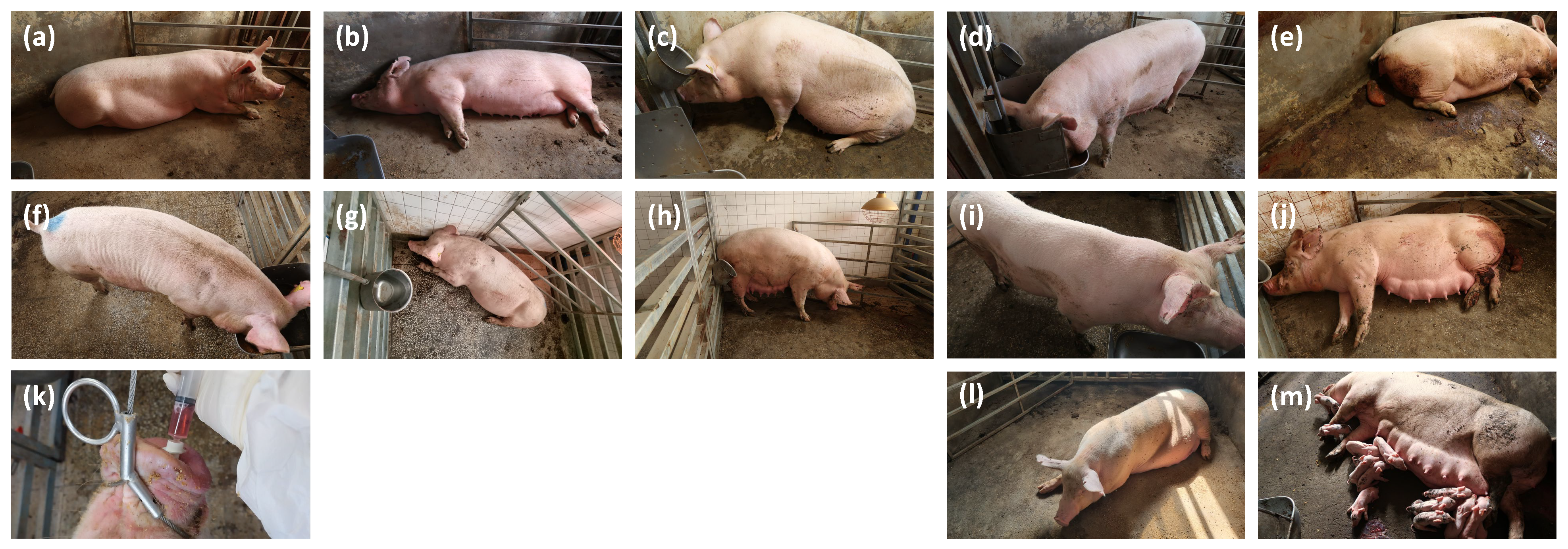

Supplement: Supporting Information 1 — Figure S1: Clinical appearance of sows from SNUVR220803-inoculated and negative control groups. Photographs of three representative sows (two from the SNUVR220803-inoculated group and one from the negative control group) taken at various days postinoculation (dpi). (a–e) A sow that aborted at 110 days of gestation. (f–j) A sow that aborted at 111 days of gestation. (k) Nasal inoculation method in a gilt under physical restraint. (l, m) Sow from the negative control group. (a, f) at 0 dpi; (b, g) at 7 dpi; (c, h) at 14 dpi; (d, i, l) at 21 dpi; and (e, j, m) at the day of delivery (24, 25, and 28 dpi, respectively). [file 1172597.f1.tiff]

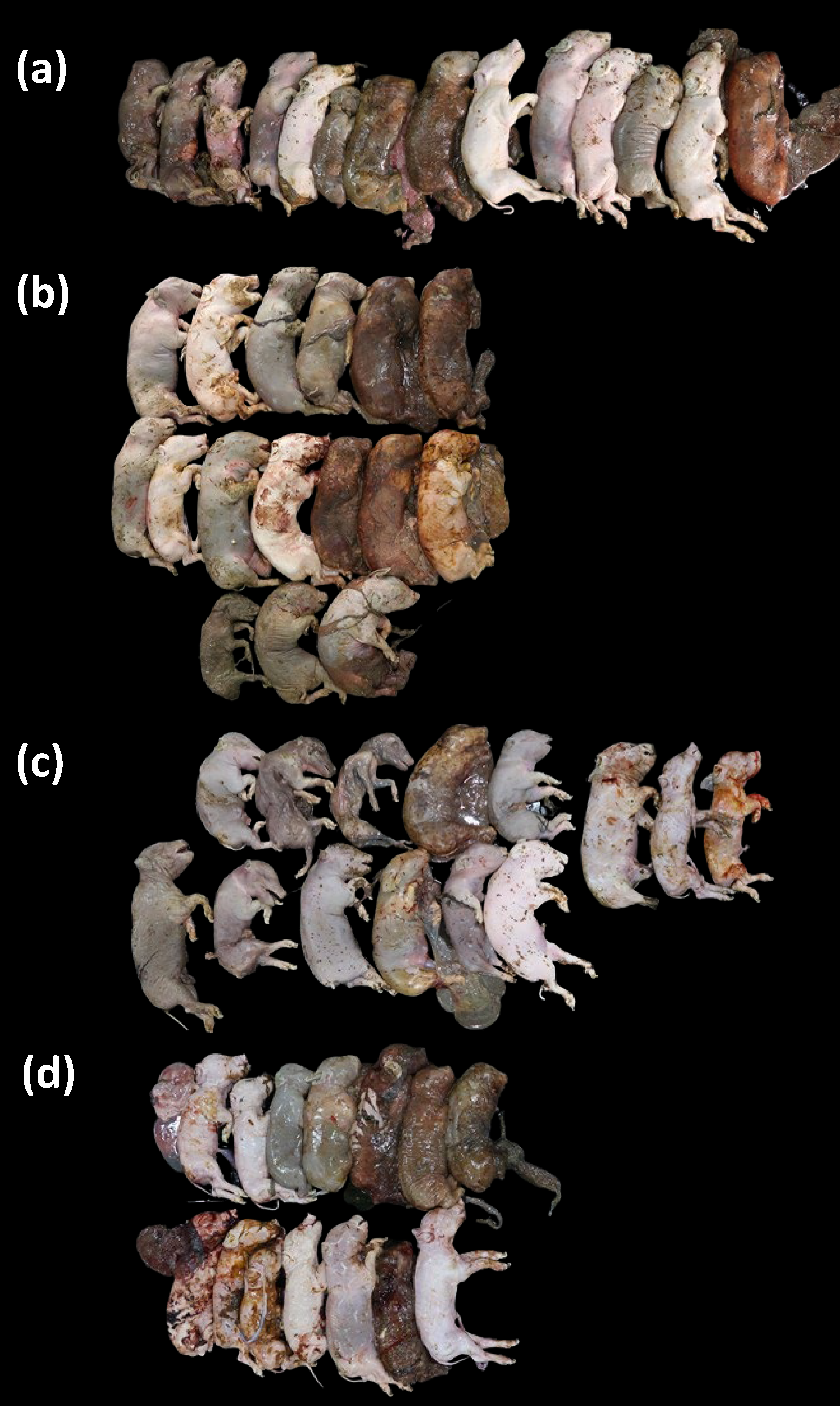

Supplement: Supporting Information 2 — Figure S2: Gross appearance of expelled or stillborn piglets from SNUVR220803-inoculated gilts. Photographs show aborted or stillborn piglets at (a) 109, (b) 110, (c) 111, and (d) 112 days of gestation. [file 1172597.f2.tiff]
